# Risk factors for aortic disease combined with coronary artery disease and the significance of preoperative coronary assessment in endovascular aortic repair

**DOI:** 10.3389/fcvm.2026.1967015

**Published:** 2026-10-09

**Authors:** Yuping Lei, Mingduo Zhang, Mengmeng Wang, Xuewen Qi, Yuzeng Xue, Yong Yang, Hang Gao, Zhongzhi Liu, Zebo Xiu

**Affiliations:** 1 Department of Cardiology, Liaocheng People’s Hospital, Liaocheng, China; 2 Medical Integration and Practice Center, Shandong University, Jinan, China; 3 Department of Cardiology, Beijing Anzhen Hospital, Capital Medical University, Beijing, China; 4 School of Clinical Medicine, Shandong Second Medical University, Weifang, China

**Keywords:** aortic disease, coronary artery disease, endovascular aortic repair, preoperative assessment, risk factors

## Abstract

**Objective:**

The clinical utility of preoperative coronary assessment in patients undergoing endovascular aortic repair (EVAR) remains uncertain. This study aims to identify risk factors for aortic disease combined with coronary artery disease (CAD) and evaluate the impact of preoperative coronary assessment on in-hospital adverse events.

**Methods:**

We conducted a single-center retrospective cohort study of patients undergoing elective EVAR at Beijing Anzhen Hospital (January 2021–June 2023). Participants were stratified by coronary assessment status within 12 months pre-surgery. Intergroup comparisons used two-independent samples *t*-tests and chi-square tests. In-hospital adverse events were a composite endpoint (all-cause mortality, acute myocardial infarction, acute heart failure, stroke, acute kidney injury, bleeding events, endoleak). Univariate logistic regression identified potential risk factors; variables with *P* < 0.10 were entered into multivariate logistic regression.

**Results:**

A total of 1,139 patients were included, of whom 385 (33.8%) underwent preoperative coronary assessment. Among assessed patients, 278 with CAD received tailored management, including revascularization or optimized medical therapy. CAD risk varied across aortic disease subtypes (*P* = 0.005); abdominal aortic aneurysm (AAA) patients had elevated risk (*OR* = 2.113, *P* = 0.027). Age (*OR* = 1.036, *P* = 0.014) and hyperlipidemia (*OR* = 2.652, *P* < 0.001) were independent CAD risk factors. The non-assessment group had higher in-hospital adverse event rates (6.0% vs. 2.6%, *P* = 0.012), primarily due to major adverse cardiovascular events (MACE) (3.4% vs. 1.3%, *P* = 0.035). Preoperative assessment was associated with reduced adverse event risk; non-assessed patients had a 277.9% increased risk (*OR* = 3.779, *P* = 0.002).

**Conclusion:**

AAA patients have a substantially elevated risk of concurrent CAD. Comprehensive preoperative coronary evaluation before EVAR optimizes medical management and enables appropriate revascularization, enhancing procedural safety and reducing adverse cardiovascular events.

## 1 Introduction

The co-occurrence of aortic diseases and coronary artery disease (CAD) has emerged as a significant clinical concern, particularly in geriatric populations. The prevalence of CAD in patients with aortic aneurysm ranges from 40% to 55%, with the proportion of abdominal aortic aneurysm (AAA) combined with CAD reaching 31% to 90% (1, 2). CAD is a major factor contributing to perioperative and long-term mortality in patients with aortic aneurysm, accounting for 60% to 70% and 40% to 70% of deaths, respectively (3). In type B aortic dissection, concomitant CAD is common and serves as an independent predictor of adverse outcomes (4, 5). Similarly, aortic ulcers often coexist with CAD, and untreated CAD increases the risk of cardiovascular events (6, 7).

Coronary assessment methods, including coronary computed tomography angiography (CCTA) and coronary angiography (CAG), can effectively identify coronary lesions, facilitating the implementation of revascularization strategies such as percutaneous coronary intervention (PCI) or coronary artery bypass grafting (CABG) when indicated, alongside the optimization of medical therapy. Evidence from prior studies has demonstrated that coronary assessment prior to open surgery for AAA significantly enhances surgical safety and reduces the incidence of postoperative cardiovascular and cerebrovascular events (8). Such preoperative coronary assessment is critical for guiding surgical decision-making, predicting postoperative outcomes, and improving overall treatment efficacy (9, 10). With the evolution of minimally invasive techniques, endovascular aortic repair (EVAR) has emerged as a pivotal intervention for aortic diseases. However, there is currently no relevant research or unified guidelines or consensus on whether preoperative coronary assessment for EVAR, along with optimization of medical therapy and perioperative revascularization based on the assessment results, can reduce in-hospital adverse events and improve patient prognosis.

This study, based on a large sample of cases from Beijing Anzhen Hospital affiliated with Capital Medical University, aims to analyze the risk factors for aortic diseases combined with CAD and explore the impact of preoperative coronary assessment for EVAR on the occurrence of in-hospital adverse events through a retrospective cohort study, thereby contributing to the development of clinical management protocols.

## 2 Methods

### 2.1 Study subjects

This study employed a retrospective cohort design. As shown in Figure 1, 1,186 patients with aortic aneurysm, aortic dissection, or aortic ulcer who underwent elective EVAR at Beijing Anzhen Hospital, Capital Medical University from January 2021 to June 2023 were included. Following the application of exclusion criteria, 47 patients were excluded, comprising 9 who underwent cardiac valve surgery, 2 who received pericardial windowing, 2 who underwent thoracoscopic lobectomy, 1 who underwent partial carotid resection with anastomosis, 1 who underwent sigmoidectomy, 28 with ruptured aortic aneurysm, and 4 with ruptured aortic dissection aneurysm. Consequently, 1,139 patients were included in the final analysis. Among them, 385 patients (33.8%) underwent coronary artery assessment (CCTA or CAG) within 12 months prior to EVAR, forming the assessment group. All CAD-related assessment results were confirmed via patient referral records or images and reports from coronary artery evaluations. Within this group, 278 patients (24.4%) diagnosed with CAD received tailored perioperative management based on their overall clinical status, comorbidities, coronary lesion characteristics, and patient preferences: 62 (5.4%) underwent PCI, 18 (1.6%) underwent CABG, and 198 (17.4%) were managed with optimized medical therapy. The remaining 107 patients (9.4%) without CAD did not receive specific interventions. In contrast, 754 patients (66.2%) who did not undergo coronary artery assessment within the 12 months prior to EVAR constituted the non-assessment group. Among them, 177 patients (15.5%) with a documented history of CAD continued their pre-existing medication regimens, while the remaining 577 patients (50.7%) with indeterminate coronary status received no specialized treatment.

**Figure 1:**
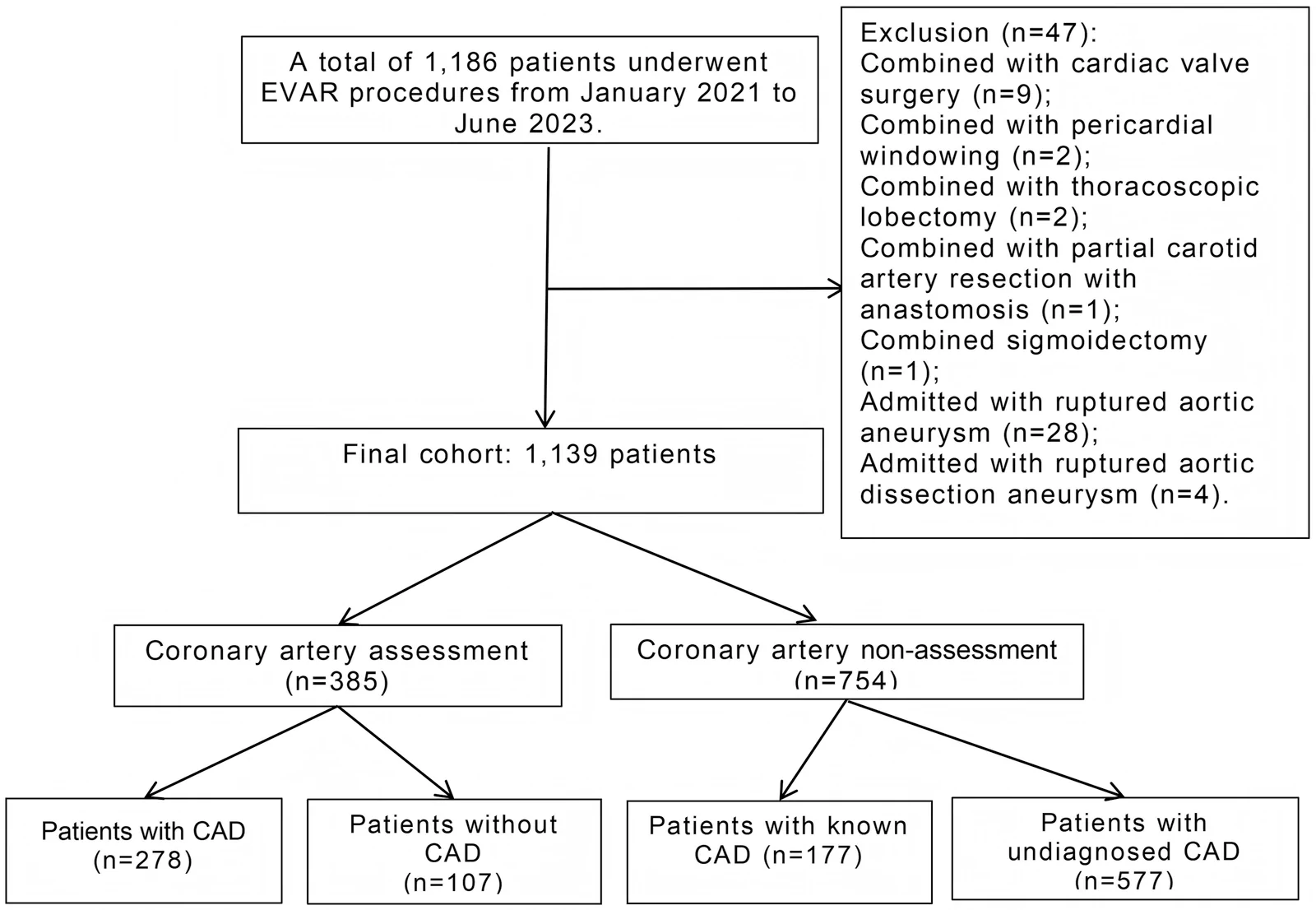
The flowchart of patient selection for the study.

### 2.2 Study endpoints and definitions

This study systematically collected and analyzed clinical, surgical, and perioperative data to investigate risk factors associated with aortic disease concurrent with CAD, as well as the influence of preoperative coronary artery assessment on in-hospital adverse events in patients undergoing EVAR. Study variables included demographic and clinical factors such as age, gender, smoking history, body mass index (BMI), hypertension, hyperlipidemia, diabetes, history of cerebral infarction, peripheral artery disease (PAD), chronic kidney disease (CKD), chronic obstructive pulmonary disease (COPD), and prior coronary revascularization (PCI or CABG). Perioperative metrics encompassed intensive care unit (ICU) duration, mechanical ventilation time, total hospital stay, and functional status at discharge, assessed using the activities of daily living (ADL) scale. Data were obtained through medical record reviews and patient follow-up to ensure accuracy and completeness. This study complied with the Declaration of Helsinki and was approved by the Institutional Ethics Committee of Beijing Anzhen Hospital, Capital Medical University (Approval No. KS2025270). Due to the retrospective nature and lack of personally identifiable information, the need for additional written informed consent was waived.

The primary endpoint was defined as in-hospital adverse events, which included any complications occurring within 30 days postoperatively or during the hospitalization period if it extended beyond 30 days. These adverse events comprised major adverse cardiovascular events (MACE), acute kidney injury (AKI), bleeding, and endoleak. Specifically, MACE was categorized as all-cause mortality, acute myocardial infarction (AMI), acute heart failure (AHF), or cerebral infarction.

### 2.3 EVAR combined with supra-aortic branch reconstruction

EVAR was performed in accordance with standard procedure. In cases where the lesion involves the aortic arch and its branches, and conventional EVAR lacks sufficient anchoring zones, EVAR is combined with either supra-aortic branch bypass grafting or supra-aortic branch stent placement. Supra-aortic branch bypass grafting reconstructs blood flow channels using artificial vessels, such as right common carotid artery-left common carotid artery-left subclavian artery bypass or left common carotid artery-left subclavian artery bypass. Supra-aortic branch stent placement maintains vessel patency during endovascular repair through techniques including parallel stenting (chimney, sandwich, or periscope techniques), fenestration (*in situ* or pre-fenestration), and branched stent placement.

### 2.4 Statistical analysis

Statistical analyses were conducted using SPSS 27.0 software (SPSS Inc., Chicago, IL, USA). Continuous variables are presented as mea*n* ± standard deviation (SD), while categorical variables are expressed as frequencies and percentages. Intergroup comparisons were performed using independent samples t-tests for continuous variables and chi-square tests for categorical variables. Univariate logistic regression models were employed to identify potential risk factors for adverse events during hospitalization. Clinical variables with *P* < 0.10 in univariate analysis were included in multivariate logistic regression models to determine whether preoperative coronary assessment constituted an independent risk factor for adverse events. We combine preoperative coronary artery assessment and CAD status into a single categorical predictor variable. This approach stratifies patients into distinct groups based on their clinical characteristics, and yields more interpretable results than using separate variables or incorporating interaction terms. A significance level of *P* < 0.05 was applied.

## 3 Results

### 3.1 Baseline clinical characteristics

According to the inclusion and exclusion criteria, a cohort of 1,139 patients undergoing elective EVAR was enrolled in this study. The cohort comprised 385 patients (33.8%) in the coronary assessment group and 754 patients (66.2%) in the non-assessment group. Comparative analysis of baseline clinical characteristics between the two groups is presented in Table 1. Patients in the coronary assessment group exhibited a significantly higher mean age compared to those in the non-assessment group (65.9 ± 8.4 vs. 60.9 ± 12.2, *P* < 0.001). The incidence of hyperlipidemia (59.2% vs. 37.8%, *P* < 0.001), diabetes mellitus (17.7% vs. 11.4%, *P* = 0.003), PAD (21.6% vs. 13.9%, *P* = 0.001), and history of coronary revascularization (24.4% vs. 8.2%, *P* < 0.001) was significantly higher in the assessment group than in the non-assessment group.

**Table 1:** Comparison of baseline clinical factors between the assessment and non-assessment groups.

Characteristics	The Non-Assessment Group (n (n%))	The Assessment Group（n (n%)）	*P*-value
Aortic Disease Type			<0.001
TAA	70 (9.3)	53 (13.8)	
AAA	262 (34.7)	200 (51.9)	
Aortic Dissection	290 (38.5)	53 (13.8)	
Aortic Ulcer	132 (17.5)	79 (20.5)	
Surgical Approach			<0.001
Abdominal EVAR	285 (37.8)	210 (54.5)	
Thoracic EVAR	346 (45.9)	108 (28.1)	
EVAR Combined with Supra-aortic Branch Bypass Grafting	79 (10.5)	43 (11.2)	
EVAR Combined with Supra-aortic Branch Stenting	44 (5.8)	24 (6.2)	
Gender			0.620
Female	108 (14.3)	51 (13.2)	
Male	646 (85.7)	334 (86.8)	
Age (years) [Mean (SD)]	60.9 (12.2)	65.9 (8.4)	<0.001
Smoking History	456 (60.5)	239 (62.1)	0.600
BMI [Mean (SD)]	25.7 (3.6)	25.7 (3.1)	0.972
Hypertension	609 (80.8)	303 (78.7)	0.409
Hyperlipidemia	285 (37.8)	228 (59.2)	<0.001
Diabetes Mellitus	86 (11.4)	68 (17.7)	0.003
History of Cerebral Infarction	73 (9.7)	52 (13.5)	0.051
PAD	105 (13.9)	83 (21.6)	0.001
CKD	44 (5.8)	25 (6.5)	0.660
Prior Coronary Revascularization	62 (8.2)	99 (25.7)	<0.001

TAA, thoracic aortic aneurysm; AAA, abdominal aortic aneurysm; EVAR, endovascular aortic repair; SD, standard deviation; BMI, body mass index; PAD, peripheral artery disease; CKD, chronic kidney disease.

The classification of aortic diseases was as follows: thoracic aortic aneurysm (TAA) in 123 cases (10.8%), AAA in 462 cases (40.6%), aortic dissection in 343 cases (30.1%), and aortic ulcer in 211 cases (18.5%). The proportion of TAA (13.8% vs. 9.3%, *P* = 0.021) and AAA (51.9% vs. 34.7%, *P* < 0.001) was significantly higher in the assessment group than in the non-assessment group; the proportion of aortic dissection was higher in the non-assessment group (38.5% vs. 13.8%, *P* < 0.001). This suggests that clinical practice tends to conduct preoperative coronary assessment for patients with TAA and AAA, whereas such assessment is less frequently carried out for patients with aortic dissection.

By surgical approach, patients included 495 cases (43.4%) of abdominal EVAR, 454 cases (39.9%) of thoracic EVAR, 122 cases (10.7%) of EVAR combined with supra-aortic branch bypass grafting, and 68 cases (6.0%) of EVAR combined with supra-aortic branch stenting. Abdominal EVAR was more prevalent in the assessment group (54.5% vs. 37.8%, *P* < 0.001), while thoracic EVAR showed higher incidence in the non-assessment group (45.9% vs. 28.1%, *P* < 0.001).

### 3.2 Comparison of adverse events and perioperative outcomes during hospitalization

As shown in Table 2, adverse events occurred in 55 patients (4.8%) during hospitalization, including mortality (*n* = 8), AMI (*n* = 11), AHF (*n* = 7), cerebral infarction (*n* = 10), AKI (*n* = 10), bleeding events (*n* = 14), type I endoleak (*n* = 11), and type II endoleak (*n* = 13). A significantly higher incidence of adverse events was observed in the unevaluated group compared to the evaluated group (6.0% vs. 2.6%, *P* = 0.012). This disparity was predominantly attributable to MACE, which exhibited a significantly greater occurrence in the unevaluated cohort (3.4% versus 1.3%, *P* = 0.035). No statistically significant differences were detected between groups for other adverse event categories.

**Table 2:** Comparison of adverse events and perioperative outcomes during hospitalization.

Characteristics	The Non-Assessment Group (n (n%))	The Assessment Group（n (n%)）	*P*-value
In-Hospital Adverse Events	45 (6.0)	10 (2.6)	0.012
MACE	26 (3.4)	5 (1.3)	0.035^#^
Mortality	7 (0.9)	1 (0.3)	0.366^#^
AMI	10 (1.3)	1 (0.3)	0.155^#^
AHF	5 (0.7)	2 (0.5)	0.915^#^
Cerebral Infarction	8 (1.1)	2 (0.5)	0.555^#^
AKI	7 (0.9)	3 (0.8)	0.938^#^
Bleeding Events	9 (1.2)	5 (1.3)	0.895^#^
Type I Endoleak	10 (1.3)	1 (0.3)	0.155^#^
Type II Endoleak	12 (1.6)	1 (0.3)	0.088^#^
ICU Duration (days) [Mean (SD)]	0.6 (2.3)	0.5 (1.6)	0.500
Mechanical Ventilation Time (hours) [Mean (SD)]	6.2 (45.3)	5.1 (17.7)	0.643
Discharge ADL Score [Mean (SD)]	83.0 (19.2)	88.7 (16.1)	<0.001
Hospital Stay (days) [Mean (SD)]	10.1 (9.5)	14.0 (7.9)	<0.001

^#^Fisher exact probability test. MACE, major adverse cardiovascular events; AMI, acute myocardial infarction; AHF, acute heart failure; AKI, acute kidney injury; ICU, intensive care unit; SD, standard deviation; ADL, activities of daily living.

Regarding perioperative outcomes, the length of hospital stay was significantly longer in the assessment group than in the non-assessment group (14.0 ± 7.9 days vs. 10.1 ± 9.5 days, *P* < 0.001), suggesting that preoperative coronary examination and management may have prolonged the hospital stay. The discharge ADL score was significantly higher in the assessment group than in the non-assessment group (88.7 ± 16.1 vs. 83.0 ± 19.2, *P* < 0.001). No statistically significant intergroup differences were observed in ICU duration (0.5 ± 1.6 days vs. 0.6 ± 2.3 days, *P* = 0.130) or mechanical ventilation time (5.1 ± 17.7 h vs. 6.2 ± 45.3 h, *P* = 0.335).

### 3.3 Risk factor analysis for aortic disease combined with CAD

As presented in Table 3, this study employed univariate and multivariate logistic regression analyses to identify independent predictors of aortic diseases combined with CAD. Initial univariate analysis revealed that aortic disease classification, age, hyperlipidemia, and diabetes mellitus were significantly associated with the complication of CAD (*P* < 0.10). Subsequently, variables demonstrating statistical significance in the univariate analysis were incorporated into a multivariate logistic regression model. Multivariate analysis revealed that the risk of CAD exhibited significant variation across different aortic disease subtypes (*P* = 0.005), with AAA demonstrating a particularly elevated risk (OR = 2.113, 95% CI: 1.091−4.092, *P* = 0.027). Furthermore, advancing age (OR = 1.036, 95% CI: 1.007−1.066, *P* = 0.014) and hyperlipidemia (OR = 2.652, 95% CI: 1.680−4.186, *P* < 0.001) were independently associated with increased CAD risk in this patient population.

**Table 3:** Univariate and multivariate logistic regression analysis of risk factors for aortic diseases with concomitant CAD.

Characteristics	Univariate Analysis		Multivariate Analysis	
	OR (95% CI)	*P* Value	OR (95% CI)	*P* Value
Aortic Disease Type		<0.001		0.005
TAA	1		1	
AAA	2.801 (1.492–5.259)	0.001	2.113 (1.091–4.092)	0.027
Aortic Dissection	0.643 (0.313–1.320)	0.229	0.800 (0.376–1.700)	0.561
Aortic Ulcer	1.123 (0.568–2.221)	0.765	0.936 (0.458–1.913)	0.856
Gender		0.170		
Female	1			
Male	1.502 (0.840–2.684)			
Age (years)	1.059 (1.032–1.088)	<0.001	1.036 (1.007–1.066)	0.014
Smoking History		0.526		
No	1			
Yes	1.149 (0.748–1.765)			
BMI	1.015 (0.949–1.085)	0.671		
Hypertension		0.301		
No	1			
Yes	1.296 (0.793–2.119)			
Hyperlipidemia		<0.001		<0.001
No	1		1	
Yes	3.342 (2.163–5.166)		2.652 (1.680–4.186)	
Diabetes Mellitus		0.084		0.368
No	1		1	
Yes	1.734 (0.928–3.239)		1.356 (0.699–2.631)	

TAA, thoracic aortic aneurysm; AAA, abdominal aortic aneurysm; BMI, body mass index; OR, odds ratio; CI, confidence interval.

### 3.4 Risk factor analysis for in-hospital adverse events

The univariate and multivariate logistic regression analyses, as presented in Table 4, demonstrated that preoperative coronary assessment prior to EVAR was significantly associated with reduced risk of in-hospital adverse events. Compared to patients with assessed CAD, those without assessment had a 277.9% increased risk of adverse events (OR = 3.779, 95% CI: 1.645−8.681, *P* = 0.002). Regarding surgical approaches, complex procedures involving supra-aortic branches were associated with significantly elevated risks of adverse events. Specifically, EVAR combined with supra-aortic branch artificial vascular bypass (OR = 5.498, 95% CI: 2.381−12.697, *P* < 0.001) and EVAR with supra-aortic branch stent placement (OR = 2.682, 95% CI: 1.033−6.961, *P* = 0.043) demonstrated substantially higher risks compared to standard abdominal EVAR procedures.

**Table 4:** Univariate and multivariate logistic regression analysis of risk factors for perioperative adverse events.

Characteristics	Univariate Analysis		Multivariate Analysis	
	OR (95% CI)	*P* Value	OR (95% CI)	*P* Value
Preoperative Coronary Assessment, CAD		<0.001		0.001
Yes, Yes	1		1	
No, Yes	3.808 (1.692–8.567)	0.001	3.779 (1.645–8.681)	0.002
Yes, No	0.282 (0.035–2.253)	0.233	0.251 (0.031–2.046)	0.197
No, No	1.354 (0.623–2.940)	0.444	1.416 (0.623–3.219)	0.407
Aortic Disease Type		0.615		
TAA	1			
AAA	0.583 (0.247–1.374)	0.217		
Aortic Dissection	0.796 (0.337–1.881)	0.603		
Aortic Ulcer	0.791 (0.309–2.022)	0.624		
Surgical Approach		0.002		<0.001
Abdominal EVAR	1		1	
Thoracic EVAR	1.031 (0.525–2.026)	0.930	1.698 (0.792–3.640)	0.173
EVAR Combined with Supra-aortic Branch Bypass Grafting	3.161 (1.503–6.645)	0.002	5.498 (2.381–12.697)	<0.001
EVAR Combined with Supra-aortic Branch Stenting	3.041 (1.221–7.576)	0.017	2.682 (1.033–6.961)	0.043
Gender		0.505		
Female	1			
Male	1.342 (0.565–3.187)			
Age (years)	1.006 (0.981–1.031)	0.648		
Smoking History		0.469		
No	1			
Yes	0.817 (0.473–1.411)			
BMI	0.984 (0.908–1.066)	0.697		
Hypertension		0.740		
No	1			
Yes	1.126 (0.559–2.271)			
Hyperlipidemia		0.536		
No	1			
Yes	1.187 (0.690–2.040)			
Diabetes Mellitus		0.069		0.137
No	1		1	
Yes	1.851 (0.953–3.595)		1.696 (0.846–3.401)	
History of Cerebral Infarction		0.387		
No	1			
Yes	1.407 (0.649–3.050)			
PAD		0.012		0.054
No	1		1	
Yes	2.175 (1.189–3.981)		2.043 (0.988–4.222)	
CKD		0.039		0.147
No	1		1	
Yes	2.404 (1.045–5.532)		1.921 (0.794–4.648)	
Prior Coronary Revascularization		0.483		
No	1			
Yes	0.734 (0.309–1.742)			

CAD, coronary artery disease; TAA, thoracic aortic aneurysm; AAA, abdominal aortic aneurysm; EVAR, endovascular aortic repair; BMI, body mass index; PAD, peripheral artery disease; CKD, chronic kidney disease; OR, odds ratio; CI, confidence interval.

## 4 Discussion

This study employed a retrospective analysis to investigate the risk factors associated with the co-occurrence of aortic disease and CAD. The findings revealed significant variations in risk profiles among different types of aortic disease when combined with CAD. Specifically, AAA demonstrated a markedly higher risk of co-occurrence with CAD compared to other aortic pathologies. Age and hyperlipidemia were identified as independent risk factors for this comorbidity. Furthermore, the study assessed the impact of coronary assessment before EVAR on in-hospital adverse events. The findings demonstrated a significantly elevated incidence of adverse events in the non-assessment group relative to the assessment group, with MACE constituting the primary contributing factor. These results highlight the critical role of preoperative coronary evaluation in reducing perioperative risks during EVAR.

The prevalence of AAA in CAD patients substantially exceeds that in the general population, ranging from 9.5% to 18% in male patients, while the comorbidity rate of CAD in AAA patients reaches 31% to 90%, suggesting a bidirectional relationship between these conditions (11). Our findings corroborate this association. Unlike TAA, which are predominantly influenced by genetic factors, AAA primarily results from degenerative changes, sharing pathophysiological mechanisms with CAD, including atherosclerosis, chronic inflammation, and vascular remodeling (12). Aortic atherosclerosis contributes to reduced vascular wall elasticity, facilitating vascular dilation and subsequent AAA formation (13). Additionally, chronic inflammatory responses may induce arterial endothelial cell damage and alter local hemodynamics, further promoting AAA development (14). Our study aligns with previous research in identifying age and hyperlipidemia as risk factors for the co-occurrence of aortic disease and CAD (15, 16). Advanced age is associated with increased susceptibility to aortic and coronary artery pathologies, and elderly patients often experience more complications and prolonged recovery periods when managing this comorbidity (3, 17). Hyperlipidemia exacerbates arteriosclerosis, thereby elevating the risk of both aortic disease and CAD.

Previous studies have demonstrated that comprehensive coronary assessment through CCTA or CAG prior to open aortic surgery can effectively identify coronary lesions, predict postoperative cardiovascular and cerebrovascular complications, and guide anesthetic and surgical strategies (18, 19). In patients with significant coronary lesions undergoing aortic surgery, perioperative revascularization significantly reduces surgical risk (20, 21) and has a positive impact on long-term survival outcomes (22–24).

The advent and widespread adoption of EVAR technology has prompted ongoing debate regarding the necessity of preoperative coronary assessment in cardiovascular practice. Multivariate logistic regression analysis in this study demonstrates that preoperative coronary evaluation in patients undergoing EVAR significantly decreases the occurrence of in-hospital adverse events, with a particularly pronounced reduction in MACE. Although patients who received preoperative assessment had higher baseline risks, their observed better clinical outcomes may reflect the role of risk stratification and individualized treatment strategies guided by preoperative coronary assessment; however, the possibility of residual confounding cannot be ruled out. These findings suggest that preoperative coronary assessment enables accurate quantification of coronary stenosis severity, facilitates optimization of medical management and perioperative revascularization in CAD patients, thereby enhancing procedural safety. Supporting evidence indicates that coronary assessment not only informs surgical decision-making for both open aortic aneurysm repair and EVAR but also predicts postoperative recovery and reduces ICU duration (3).

Notably, 66.2% of the patients in the present study cohort did not undergo preoperative coronary assessment. European guidelines recommend that patients undergoing elective vascular surgery, including EVAR, complete a preoperative cardiac evaluation (25); however, real-world data reveal a gap between these guidelines and clinical practice (26). Several factors contribute to this discrepancy: the absence of standardized operating procedures, resulting in individualized protocols often formulated based on patient conditions and team experience; a tendency among surgeons to emphasize coronary assessment prior to open surgery while paying insufficient attention to preoperative evaluation for EVAR, which occasionally leads to direct surgical intervention due to the perceived low risk of CAD; and the perception among attending physicians that further evaluation is unnecessary for certain patients with confirmed CAD who are already receiving outpatient pharmacotherapy. The higher incidence of in-hospital adverse events in the non-assessment group underscores the importance of adhering to guidelines, highlighting the necessity to develop standardized preoperative assessment protocols and to enhance awareness of the risks associated with concomitant CAD.

Currently, the optimal surgical sequence for combined EVAR and coronary revascularization remains controversial. Contemporary clinical practice favors an individualized approach to surgical sequencing based on the severity of CAD, the risk of aortic aneurysm rupture, and the surgical team's experience. In the presence of severe CAD, such as acute coronary syndrome, coronary revascularization is recommended as the initial intervention to improve myocardial perfusion and stabilize cardiac function, thereby establishing safer hemodynamic conditions for subsequent aortic surgery (13). Conversely, when the risk of aneurysm rupture is high, addressing the aortic pathology first is advocated to prevent the progression or rupture of the aortic lesion during coronary revascularization (27). Furthermore, some clinical teams have attempted simultaneous EVAR and coronary revascularization, aiming to resolve both pathologies in a single operation while reducing hospital stay and medical costs; however, the complexity and safety of this approach require further validation (23).

Furthermore, the combination of EVAR with supra-aortic branch reconstruction is associated with a marked increase in adverse event risk, attributable to disease severity and procedural complexity. The management of aortic arch pathologies continues to present substantial clinical challenges. However, advancements in medical technology, accumulation of clinical evidence, and innovations in medical devices are driving a paradigm shift from traditional open surgical approaches toward hybrid and total endovascular repair techniques, which are anticipated to further reduce surgical morbidity in the future.

This study underscores the critical role of preoperative coronary assessment in patients undergoing EVAR, yet several limitations warrant consideration. First, as a retrospective cohort study, the research is susceptible to selection bias and the unassessed/undiagnosed CAD subgroup includes patients with indeterminate CAD status, encompassing both individuals without CAD and those with undetected CAD, which may introduce misclassification bias. Second, the single-center nature of the sample may limit the generalizability of findings to diverse patient populations across different regions or healthcare settings. Finally, we acknowledge that multiple endpoints (MACE, AKI, bleeding events, and endoleak) were tested individually without multiplicity adjustment. Therefore, these analyses should be considered exploratory and require cautious interpretation. Consequently, future investigations should prioritize prospective, multicenter, large-scale clinical trials with pre-specified multiplicity corrections to validate these findings.

## 5 Conclusion

Patients with AAA exhibit a significantly elevated risk of CAD compared to those with other aortic pathologies. Age and hyperlipidemia were identified as independent risk factors for aortic disease combined with CAD. Preoperative coronary assessment prior to EVAR was associated with a marked reduction in the risk of in-hospital adverse events, highlighting its value in optimizing treatment strategies and enhancing surgical safety. Future research should focus on refining preoperative assessment protocols and developing tailored therapeutic approaches for high-risk patients to improve long-term outcomes.

## Data Availability

The original contributions presented in the study are included in the article/Supplementary Material, further inquiries can be directed to the corresponding author.

## References

[1] Orecchia PM Berger PW White CJ Algeo J Gomez ER McDonald PT. Coronary artery disease in aortic surgery. Ann Vasc Surg. (1988) 2:28–36. 10.1016/S0890-5096(06)60774-7 2976277

[2] Hernesniemi JA Vänni V Hakala T. The prevalence of abdominal aortic aneurysm is consistently high among patients with coronary artery disease. J Vasc Surg. (2015) 62(1):232–240.e3. 10.1016/j.jvs.2015.02.037 26115925

[3] Watanabe K Watanabe T Otaki Y Kato S Tamura H Nishiyama S. Impact of pre-operative coronary artery disease on the clinical outcomes of patients with aortic aneurysms. Heart Vessels. (2021) 36(3):308–14. 10.1007/s00380-020-01700-2 32926229

[4] Hashiyama N Goda M Uchida K Isomatsu Y Suzuki S Mo M. Stanford type B aortic dissection is more frequently associated with coronary artery atherosclerosis than type A. J Cardiothorac Surg. (2018) 13(1):80. 10.1186/s13019-018-0765-y 29945663 PMC6020428

[5] Li W Luo S Lin W Su S Xu W Hu X. Coronary artery disease as an independent predictor of short-term and long-term outcomes in patients with type-B aortic dissection undergoing thoracic endovascular repair. Front Cardiovasc Med. (2022) 9:1041706. 10.3389/fcvm.2022.1041706 36588578 PMC9795049

[6] Weiss S Sen I Huang Y Killian JM Harmsen WS Mandrekar J. Cardiovascular morbidity and mortality after aortic dissection, intramural hematoma, and penetrating aortic ulcer. J Vasc Surg. (2019) 70(3):724–731.e1. 10.1016/j.jvs.2018.12.031 30871888 PMC7477938

[7] Daimon M Miyata H Motomura N Okita Y Takamoto S Kanki S. Outcomes of thoracic aortic surgery in patients with coronary artery disease—based on the Japan adult cardiovascular surgery database. Circ J. (2019) 83(5):978–84. 10.1253/circj.CJ-18-0703 30842374

[8] Sandner SE Nolz R Loewe C Gregorich M Heinze G Andreas M. Routine preoperative aortic computed tomography angiography is associated with reduced risk of stroke in coronary artery bypass grafting: a propensity-matched analysis. Eur J Cardio-Thorac. (2020) 57 (4): 684–90. 10.1093/ejcts/ezz237 31504374

[9] Kadric N Kabil E Mujanovic E Hadziselimovic M Jahic M Rajkovic S. Operative treatment of combined aortic stenosis and coronary artery disease. Med Arch. (2015) 69(1):54–7. 10.5455/medarh.2015.69.54-57 25870480 PMC4384859

[10] Otsu M Ishizaka T Watanabe M Hori T Kohno H Ishida K. Analysis of anatomical risk factors for persistent type II endoleaks following endovascular abdominal aortic aneurysm repair using CT angiography. Surg Today. 2015 46 (1): 48–55. 10.1007/s00595-015-1115-5 25578204

[11] Takagi H Umemoto T. Coronary artery disease and abdominal aortic aneurysm growth. Vasc Med. (2016) 21(3):199–208. 10.1177/1358863X15624026 26842623

[12] Schermerhorn ML Giles KA Sachs T Bensley RP O’Malley JA Cotterill P. Defining perioperative mortality after open and endovascular aortic aneurysm repair in the US medicare population. J Am Coll Surg. (2011) 212:349–55. 10.1016/j.jamcollsurg.2010.12.003 21296011 PMC3051838

[13] Mannacio VA Mannacio L Monaco M Antignano A Giordano R Pinna GB. Safety of aortic aneurysm repair 8 weeks after percutaneous coronary intervention for coronary artery disease: a cohort study. Updates Surg. (2020) 72:1213–21. 10.1007/s13304-020-00729-2 32141047

[14] Mannacio VA Mannacio L Antignano A Monaco M Mileo E Pinna GB. Status of coronary disease and results from early endovascular aneurysm repair after preventive percutaneous coronary revascularization. J Cardiac Surg. (2021) 36:834–40. 10.1111/jocs.15305 33415770

[15] Baguet J-P Chavanon O Sessa C Thony F Lantelme P Barone-Rochette G. European society of hypertension scientific newsletter: hypertension and aortic diseases. J Hyertens. (2012) 30(2):440–3. 10.1097/HJH.0b013e32834f867a 22179093

[16] Assania R Daulay E Hasan R. Association of risk factors for coronary artery disease with the incidence of abdominal aortic calcification on abdominal CT-scan imaging in H. Adam malik general hospital. JSocMed. (2023) 2(3):52–9. 10.47353/jsocmed.v2i3.38

[17] Vänni V Hernesniemi J Turtiainen M Turtiainen J Hakala T. Screening men with coronary heart disease for abdominal aortic aneurysm: a prospective cohort study. World J Surg. (2015) 39(9):2354–8. 10.1007/s00268-015-3091-8 25975589

[18] Rhee R Gupta A Vechvitvarakul S Hoque M Ruggiero M Shih M. Risk factors predictive of unfavorable distal aortic remodeling after surgical repair of type A thoracic aortic dissection. J Cardiovasc Surg. (2019) 61 (3): 323–31. 10.23736/S0021-9509.19.10784-7 31140769

[19] Khan AS Kumar S Khatri M. Letter to the editor: preoperative evaluation of aortic calcification by computed tomography in thoracic aortic disease. J Cardiac Surg. (2022) 37(12):5693–4. 10.1111/jocs.16990 36183394

[20] Sigl M Baumann S Könemann AS Keese M Schwenke K Gerken ALH. Prognostic value of extended cardiac risk assessment before elective open abdominal aortic surgery. Herz. (2023) 49 (3): 210–8. 10.1007/s00059-023-05209-y 37789149

[21] Arpesani A Giorgetti PL Rampoldi V Bordoni MG Anguissola GB Tealdi D. Cardiological risk in patients undergoing abdominal aortic aneurysm surgery. An analysis of 523 cases. Panminerva Med. (1991) 33(1):11–6. 1876446

[22] Quintana E Bajona P Schaff HV Dearani JA Daly R Greason K. Open aortic arch reconstruction after coronary artery bypass surgery: worth the effort? Semin Thorac Cardiov. (2016) 28(1):26–35. 10.1053/j.semtcvs.2015.12.006 27568130

[23] Nardi P Rinaldi V Costanzo ML Pasqua R Loiacono F Palumbo P. Simultaneous percutaneous coronary intervention (PCI) and endovascular aneurysm repair (EVAR): a preliminary report. J Clin Med. (2024) 13:5545. 10.3390/jcm13185545 39337032 PMC11432325

[24] Sevilla N Clara A Diaz-Duran C Ruiz-Carmona C Ibañez S. Survival after endovascular abdominal aortic aneurysm repair in a population with a low incidence of coronary artery disease. World J Surg. (2016) 40:1272–8. 10.1007/s00268-015-3377-x 26711643

[25] Zettervall SL Schanzer A ESVS 2024 Clinical practice guidelines on the management of abdominal aorto-iliac artery aneurysms: a north American perspective. Eur J Vasc Endovasc. (2024) 67 (2): 187–9. 10.1016/j.ejvs.2023.10.036 37918617

[26] Eldrup-Jorgensen J Kraiss LW Chaikof EL Neal D Forbes TL. Vascular quality initiative assessment of compliance with society for vascular surgery clinical practice guidelines on the care of patients with abdominal aortic aneurysm. J Vasc Surg. (2020) 72(3):874–85. 10.1016/j.jvs.2019.10.097 31973949

[27] Ikeda S Mine T Sekine T Abe M Ito H Okada K. Evaluation of blood flow energy loss before and after endovascular aneurysm repair using four-dimensional flow MR imaging. Magn Reson Med Sci. (2026) 25(1):n/a. 10.2463/mrms.mp.2025-0031 PMC13287559 41605670

